# Correction: An Epstein-Barr virus–encoded microRNA targets PUMA to promote host cell survival

**DOI:** 10.1084/jem.2007258108072026c

**Published:** 2026-08-21

**Authors:** Elizabeth Yee-Wai Choy, Kam-Leung Siu, Kin-Hang Kok, Raymond Wai-Ming Lung, Chi Man Tsang, Ka-Fai To, Dora Lai-Wan Kwong, Sai Wah Tsao, Dong-Yan Jin

Vol. 205, No. 11 | https://doi.org/10.1084/jem.20072581 | October 6, 2008

The authors regret that an error was introduced during revision of their paper when representative bands for 5S rRNA were selected. In Fig. 2 A, bands from lanes 4 and 5 were inadvertently used in lanes 1 and 2. Lanes 1–5 have been replaced with the correct bands from the full gel. The original and corrected figures are shown here. This correction does not change the original conclusions of the article, and the figure legend remains unchanged. The HTML and PDF versions of this article have been corrected. The error remains only in print and in PDFs downloaded before August 12, 2026.

**Figure:**
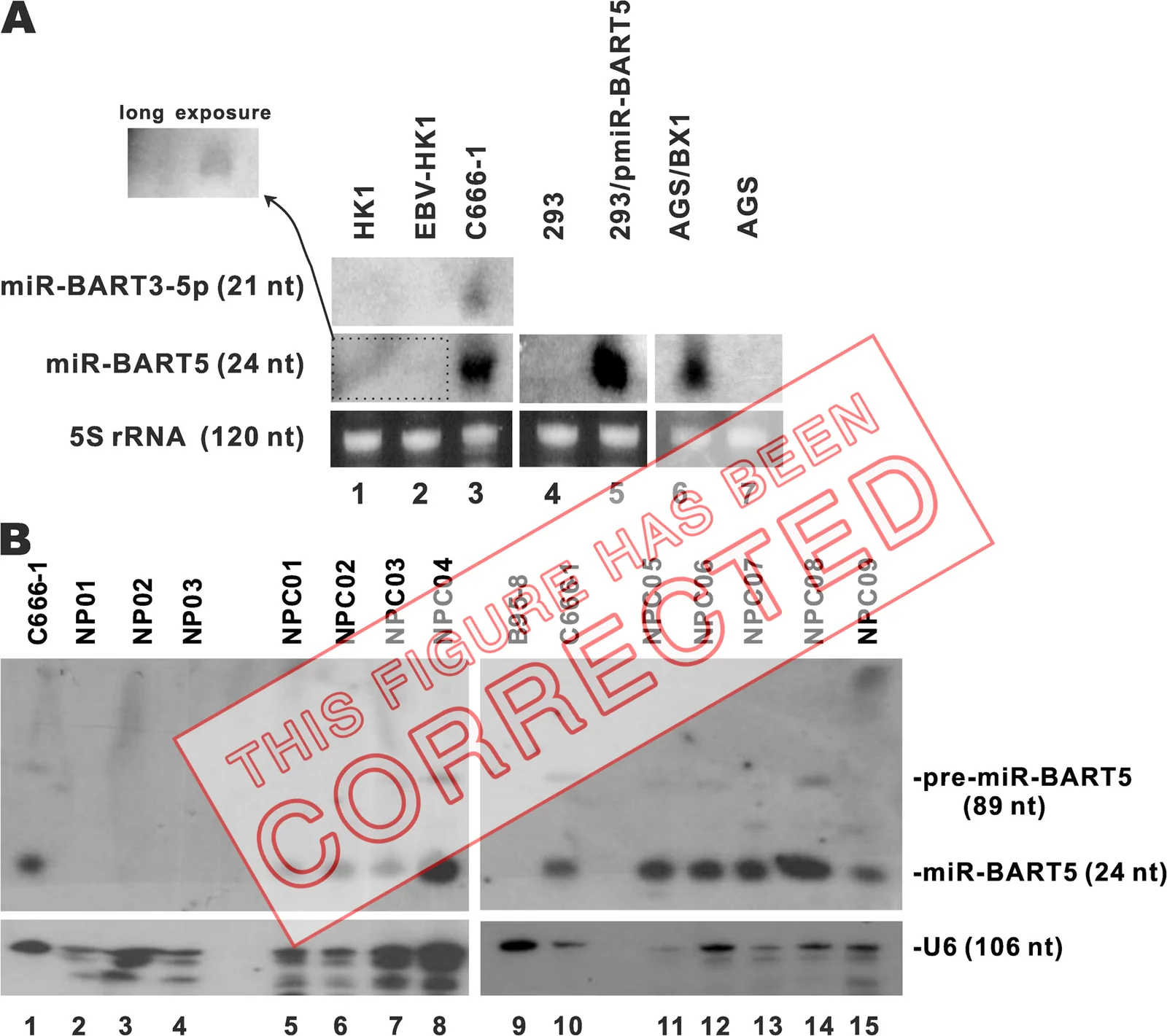


**Figure 2.**
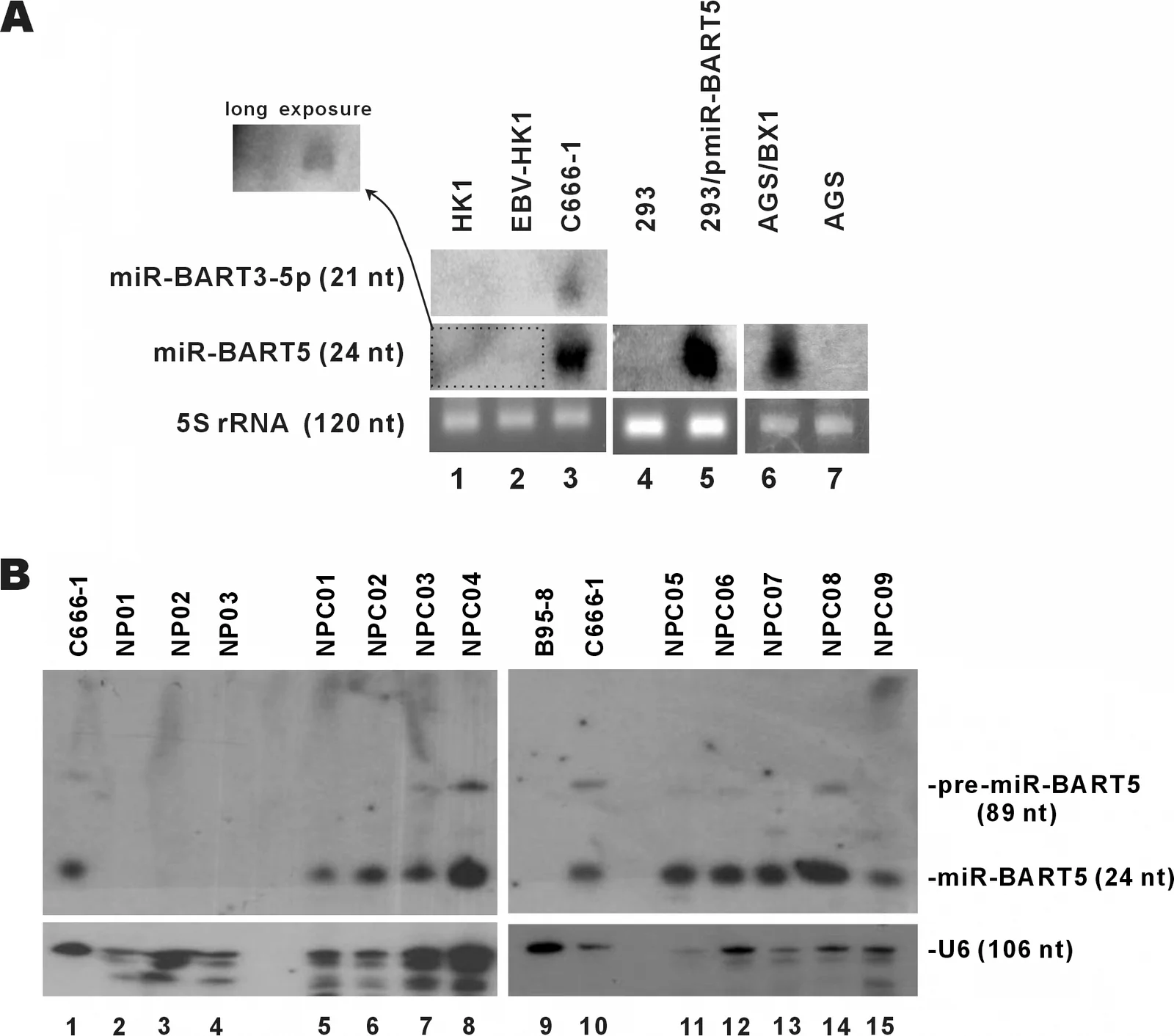
**Expression of miR-BART5 in EBV-infected epithelial cells. (A)** Northern blot analysis of EBV miRNAs in NPC and EBV-GC cell lines. HK1, HK1/EBV, and C666-1 are NPC cell lines (lanes 1–3). AGS/BX1 is an EBV-GC cell line (lane 6). Untransfected HEK293 cells (293; lane 4), HEK293 cells stably expressing miR-BART5 (293/pmiR-BART5; lane 5), and AGS cells (lane 7) were included as controls. Expression signal of miR-BART5 in HK1/EBV cells was visible only after long exposure (inset). The 5S rRNA served as a loading control. Although lanes 1–5 in the middle were from the same exposure of one experiment, lanes 6 and 7 were from another experiment. **(B)** Northern blot analysis of miR-BART5 in human NPC tissue samples. Three normal nasopharyngeal biopsies (NP01, NP02, and NP03; lanes 2–4) and nine NPC tumor samples (NPC01 to NPC09; lanes 5–8 and 11–15) were analyzed. C666-1 and latency III B95-8 lymphoblastoid cells (lanes 1, 9, and 10) were included as positive and negative controls, respectively. U6 small nuclear RNA served as a loading control.

